# Histology of PFO-associated stroke thrombus compared to iliofemoral deep vein thrombus: an explorative study

**DOI:** 10.1007/s00234-025-03693-z

**Published:** 2025-07-02

**Authors:** M. H. M. Immens, M. Stam, D. W. J. Dippel, G. J. Lycklama à Nijeholt, H. B. van der Worp, S. Jenniskens, M. J. van Rijn, F. E. de Leeuw, T. J. F.ten Cate, H. M. M. van Beusekom, Anil M. Tuladhar

**Affiliations:** 1 Donders Institute for Brain, Cognition and Behaviour, Department of Neurology, Radboud University Medical Center, Nijmegen, Netherlands; 2 Department of Cardiology, Erasmus Medical Center, Rotterdam, the Netherlands; 3 Department of Neurology, Erasmus Medical Center, Rotterdam, the Netherlands; 4 Department of Radiology, Haaglanden Medical Center, The Hague, the Netherlands; 5 Department of Neurology and Neurosurgery, University Medical Center Utrecht, Utrecht, the Netherlands; 6 Department of Radiology, Radboud University Medical Center, Nijmegen, the Netherlands; 7 Department of Vascular Surgery, Erasmus Medical Center, Rotterdam, the Netherlands; 8 Department of Cardiology, Radboud University Medical Center, Nijmegen, the Netherlands; 9 Department of Neurology, Research Institute for Medical Innovation, Radboud University Medical Centre, PO Box 9101, 6500 HB Nijmegen, the Netherlands

**Keywords:** Stroke, Patent foramen ovale, Histology

## Abstract

**Purpose:**

A patent foramen ovale (PFO) is as a cause of thrombo-embolic stroke. It is thought that the thrombus originates from the venous circulation, although this has never been proven. The histological composition of the thrombus might help to identify its origin. The aim of this exploratory pilot study is to compare the histological composition of thrombi of patients with PFO-associated stroke with venous thrombi from patients with iliofemoral deep venous thrombosis (DVT).

**Methods:**

We retrieved thrombi from the MR CLEAN Registry, a Dutch nationwide, multicenter, prospective registry of patients who underwent endovascular treatment for ischemic stroke. Furthermore DVT thrombi were obtained as fully anonymous waste material and analyzed retrospectively.

**Results:**

Thrombi were available for three patients treated for PFO-associated stroke and four patients treated for DVT. The thrombi of patients with PFO-associated stroke contained less red blood cells (RBC) and more fibrin and platelets (Fib + Plt) than those with DVT (30.2% vs. 91.3% RBC and 67.4% vs. 8.5% Fib + Plt). The PFO-associated stroke thrombi were most comparable to thrombi from the same cohort classified as cardioembolic (RBC 25.8% and 67.1% Fib + Plt). As this is a descriptive histological analysis, no definitive comparisons between different thrombi can be made.

**Conclusion:**

We observed that the composition of the three thrombi from patients with PFO-associated stroke differs from that of the four DVT thrombi in our cohort. Prospective studies are needed to determine whether thrombi in PFO-associated stroke are all similar in composition and share a similar pathophysiology with venous thrombi.

## Background

A PFO is present in about 25% of all healthy people, but may in rare instances convert to a cause of stroke [1, 2]. Because of the high prevalence of “innocent bystander PFOs”, it is crucial to separate them from stroke-causing PFOs in order to prevent unnecessary treatment of asymptomatic PFOs. The mechanism by which a PFO causes a stroke includes the passage of a thrombus, presumably originating from the venous circulation to the arterial circulation, though this has never been directly demonstrated. An alternative pathological mechanism suggests in situ thrombus formation within the PFO tunnel [3]. The histopathological composition of thrombi might reveal the actual origin of the thrombus as arterial thrombi mainly consist of fibrin and platelets (Fib + Plt), while venous thrombi mostly contain red blood cells (RBC) [4]. The aim of this exploratory pilot study is to characterize and compare the histopathology of thrombi of patients with presumed PFO-associated stroke, symptomatic iliofemoral deep venous thrombosis (DVT) and strokes of other etiologies.

## Methods - patients

### Patients with an ischemic stroke

We used data from the MR CLEAN Registry [5]. In short, the MR Clean registry is a Dutch nationwide, multicenter, prospective registry of patients who underwent endovascular treatment (EVT) for ischemic stroke between March 2014 until June 2016. For the purpose of our particular substudy we only selected patients between 18 and 60 years. After analyzing hundreds of medical discharge letters, we observed that patients diagnosed with PFO-associated stroke during initial admission were relatively young (below the age of 60). We reviewed all medical discharge letters from the MR CLEAN Registry for patients up to 65 years of age and found no applicable candidates between the ages of 60 and 65. Therefore, we did not examine medical release letters for patients older than 65, as we did not expect to identify additional suitable candidates for our study.

Stroke etiology was determined based on the diagnosis at discharge. The presence of a PFO was assessed with transthoracic echocardiography (TTE) and/or transesophageal echocardiography (TEE) following a bubble test with agitated saline. Patients with an atrial septal defect (ASD) were not included. A PFO was considered the cause of stroke if patients had undergone CT angiography of the cervical arteries to rule out atherosclerosis and had at least 24-hour cardiac rhythm monitoring to rule out atrial fibrillation. In the Netherlands, screening for DVT is not part of the standard diagnostic workup for stroke patients presenting with PFO, unless there is a clinical suspicion of DVT, which was not the case in these patients [6]. Thrombi available for histopathological analysis were analyzed.

### Patients with a DVT

Thrombi of patients with a symptomatic DVT were part of a separate cohort. They underwent percutaneous thrombectomy within the iliofemoral vein due to severe symptoms. These thrombi were obtained as fully anonymous waste material and analyzed retrospectively. The thrombi were retrieved and stored at the Erasmus Medical Center, Rotterdam, The Netherlands, for undetermined research purposes. Due to anonymization, no patient data were available.

According to Dutch law, fully anonymous waste material can be used without medical ethics approval.

### Patients with a cardioembolic or non-cardioembolic stroke

Furthermore, we looked at the analyzed thrombi from the MR CLEAN Registry, categorized as cardioembolic or non-cardioembolic. The quantification of these thrombi is described in a previous study by Hund et al., who also investigated the association between thrombus composition and stroke etiology in the same cohort [7]. 

### Processing and histopathological analysis of the thrombi

Thrombi were collected, fixed, photographed and stored in 4% buffered formaldehyde prior to paraffin embedding. DVT samples contained a large number of fragments, sometimes more than 60 fragments per patient. Hence a random set of maximally three blocks with multiple fragments were taken for histological analysis. All thrombus fragments collected during EVT were stored in one block and thus all segments were analyzed.

Samples were stained for hematoxylin-eosin as a routine stain and digitalized using the Hamamatsu scanner [5]. In short, digitized images were histologically analyzed for RBC, Fib + Plt and leukocytes using a machine learning algorithm (Orbit Image Analysis software (Orbit Image Analysis, Idorsia Ltd.). Orbit Image Analysis shows good performance as an image analysis tool compared to manual visual control [8]. The weighted average of the sections was used for data-analysis [5]. In case of missing areas of RBC due to processing artefacts, these areas were manually traced and data was then corrected for these areas. Quantitative analysis of thrombus composition was performed for all images within the same session using Orbit analysis software; hence, the analysis was by definition blinded to the source. Qualitative analysis was conducted by an experimental pathobiologist who was not blinded to the source.

### Statistical analyses

The composition of thrombi from PFO-associated stroke was compared to thrombi obtained from patients with DVT. Statistical analysis was done using GraphPad Prism 8.0.1. Data are shown as mean percentage (%) ± standard deviation. No statistical tests were performed to compare the groups due to the small number of patients. We compared the quantification of the thrombus characteristics from PFO and DVT with the analyzed cardioembolic and non-cardioembolic thrombus from the same cohort as described by Hund et al. [7]

## Results

We identified 18 patients with a PFO-associated stroke in the MR CLEAN Registry. Of these, three patients (aged 30–50 years) had thrombi available for analysis (Table 1).

**Table 1:** Description of patients with PFO-associated stroke

Patient	Gender	Age	Medical history	Cardiovascular risk factors	Event	Intravenous thrombolysis
PFO-1	Female	50 years	Tetralogy of Fallot, angina pectoris, TIA in 2009 and 2011, since 2009 a known PFO, which was not closed due to preference for best medical treatment	Dyslipidemia and smoking	Left M2 occlusion	Yes
PFO-2	Female	30 years	Migraine with aura	None	Right M1 occlusion	No
PFO-3	Female	48 years	Tonsillectomy at young age, usage of oral contraceptive pill	Dyslipidemia	Right M1 occlusion	Yes

Investigations according to national stroke guidelines did not reveal any other cause of stroke than the PFO

## Histological results

**Fig. 1:**
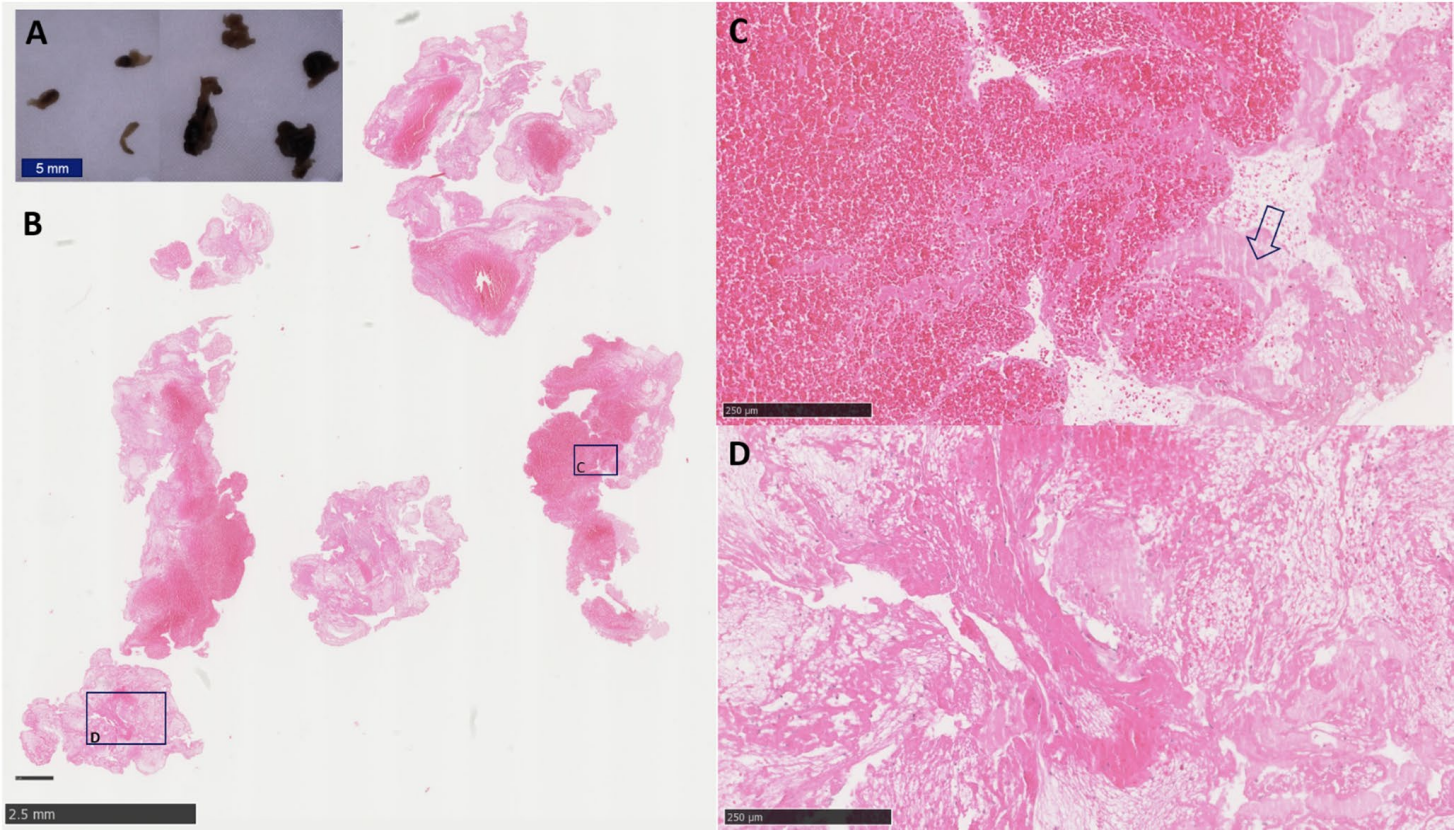
Stroke thrombus histology (PFO-2). Macroscopy (**A**) and microscopy (**B**-**D**) of thrombi retrieved for acute ischemic stroke. The diversity in thickness and length of the fragments is illustrated in the macroscopic image of the fixed thrombi (**A**). Histology (**B**-**D**) illustrates the presence of both erythrocyte rich (**C**) and fibrin rich areas (**D**). Zahn-lines observed in panel **C** (arrow), which can be seen in both venous and arterial thrombi indicate that part of the thrombus was formed under flowing blood conditions. Hematoxylin Eosin stain (**B**-**D**). Bars indicate magnification

Thrombi of PFO-associated stroke showed that they consisted for 30.2 ± 6.1% of RBC, 67.4 ± 7.3% of Fib + Plt and 2.4 ± 2.2% of leukocytes (Fig. 1).

**Fig. 2:**
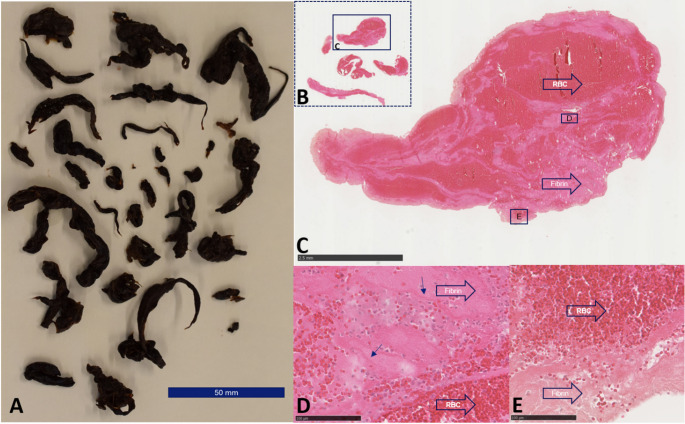
Histology of iliofemoral deep venous thrombus (DVT1). Macroscopy (**A**) and microscopy (**B**-**D**) of thrombi retrieved from patients with an iliofemoral deep venous thrombosis. The diversity in thickness and length of the thrombi is illustrated in the macroscopic image of the fixed thrombi (**A**). Histology (**B**-**D**) illustrates the presence of both erythrocyte rich areas (RBC, arrowheads) and fibrin rich areas (Fibrin, arrowheads) in the center of the thrombus fragments (**C**) as well as at the edges of the fragments (**D**). Zahn-lines observed in panel **C**, which can be seen in both venous and arterial thrombi indicate that part of the thrombus was formed under flowing blood conditions. This is not specific for DVT thrombi. Hematoxylin Eosin stain (**B**-**D**). Bars indicate magnification

**Fig. 3:**
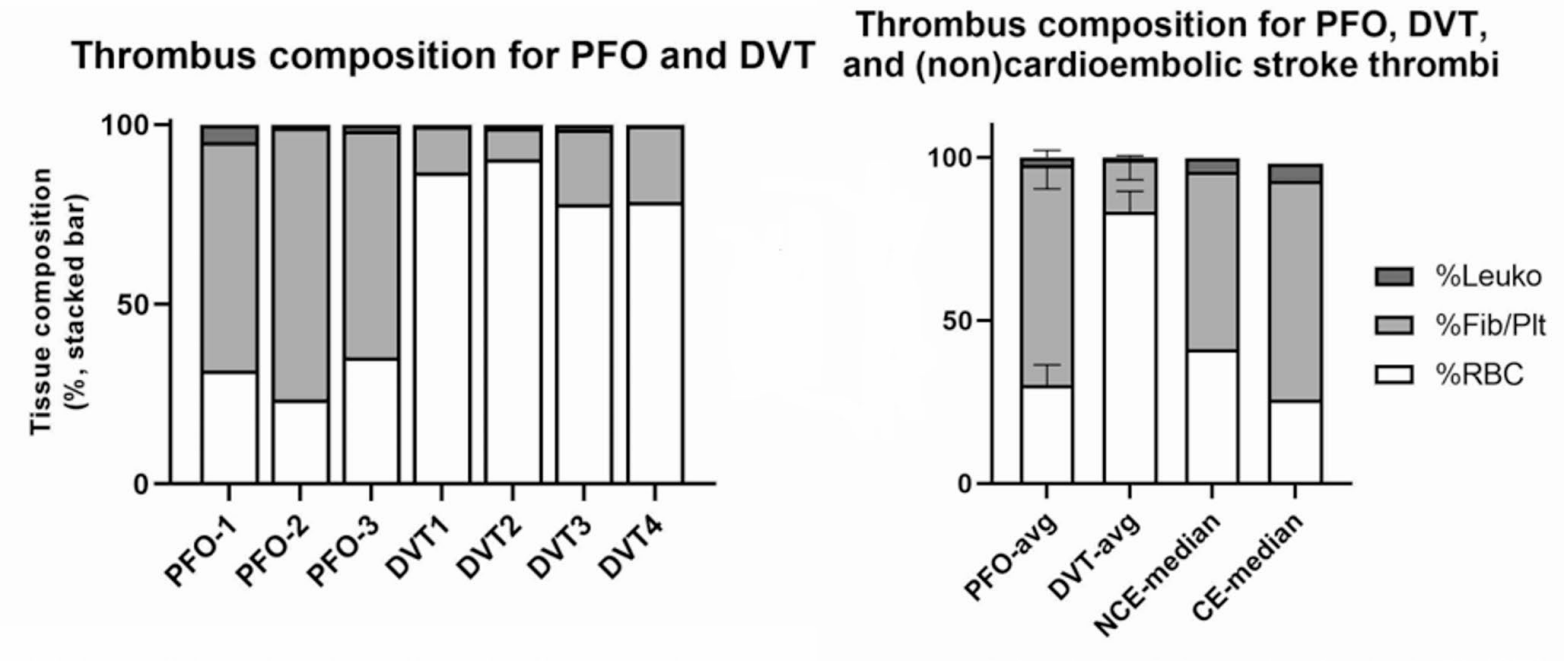
Differences in thrombus composition. Tissue composition= histological composition of the thrombus, depicted as a percentage of %Leuko= Leukocytes, %Fib/Plt= Fibrin and platelets and %RBC= Red blood cells. PFO= Patent foramen ovale thrombus, DVT= Deep venous thrombus, avg= average, NCE= non-cardioembolic, CE= cardioembolic, *Data on the groups: NCE-median and CE-median are derived from Hund et al. [7]

Thrombi of patients with DVT consisted for 83.4 ± 6.2% of RBC, 15.9 ± 6.2% of Fib + Plt and 0.7 ± 0.5% of leukocytes (Fig. 2). The thrombus composition of PFO-associated strokes was different compared to DVT. The percentage of RBC’s was lower and the Fib + Plt higher in thrombi of PFO-associated stroke compared to thrombi of patients with DVT (Fig. 3, panel 1). In contrast, the PFO-associated stroke thrombi were similar to thrombi from patients with a cardioembolic cause of stroke (RBC 25.8% and 67.1% Fib + Plt) (Fig. 3, panel 2). As this is a descriptive histological analysis, no definitive comparisons between different thrombi can be made.

## Discussion

In our study, PFO-associated stroke thrombi differed histologically from DVT thrombi and were most comparable to the composition of thrombi retrieved from patients with a different cardioembolic cause of stroke.

The correlation between DVT and PFO-associated stroke remains unclear, although the paradoxical embolism theory is widely accepted. Studies report a wide frequency of DVT between 7 and 27% in patients with a PFO-associated stroke. Based on the current literature, routine screening for DVT in PFO-associated stroke patients is not justified [9]. This approach is in contrast with the standard care for diagnosing pulmonary embolism, where routinely searching for DVT is a common practice. This appears contradictory, as the paradoxical embolism theory assumes that the thrombus causing a PFO-associated stroke shares the same pathophysiology as a pulmonary embolism. Identifying the thrombus of PFO-associated stroke is crucial, as the treatment involves percutaneous closure of the orifice and potentially discontinuing antithrombotic therapy after closure in young stroke patients [10]. Consequently, patients who are incorrectly diagnosed with a PFO-associated stroke risk receiving erroneous treatment and are exposed to potential surgical complications after closure of PFO like atrial fibrillation [11]. 

Previous research has indicated that stroke-causing thrombi exhibit a broad spectrum of composition, ranging from predominantly fibrin and platelets to primarily RBC [5, 6]. With our study, we wanted to pilot the future role of histological analyses in determining which patients could benefit from percutaneous closure. In our cohort, PFO-associated stroke thrombi did not differ from cardioembolic thrombi in terms of RBC, Fib/Plt, and Leuko composition. Other parameters, such as thrombin levels of the clot, may have the potential to distinguish PFO-associated stroke from other etiologies [12]. 

Percutaneous closure of the PFO, despite having a high number needed to treat, has been proven beneficial in preventing recurrent PFO-associated stroke. There are several potential explanation for this: 1) percutaneous closure prohibits the passage of thrombi originating from the tunnel, as is supported by Yan et al. [3], 2) percutaneous closure restores the atrial integrity and therefore the atrial function [13], or 3) alternatively, percutaneous closure may prevent thrombus originating from the venous circulation passing through the tunnel, which could not be proven by our study as the PFO-associated stroke thrombus composition differed from DVT thrombus. Another possibility could be that PFO-associated strokes have different etiologies, as mentioned above, and that some thrombi indeed originate within the venous system. Future studies are necessary to compare thrombi from PFO patients who have had a stroke and undergone mechanical thrombectomy, with and without DVT. This approach could potentially be of value in identifying patients who would benefit from a PFO closure.

Our results are in contradiction with the results of another study that reported a significantly higher percentage of RBC and lower percentage of Fib + Plt in thrombi of patients with a PFO and with a cryptogenic stroke compared to patients without a PFO and with a cryptogenic stroke [14]. However, that study does not report whether these PFOs were considered the cause of stroke or if they were incidental findings. In the previous study, patients with a PFO, cryptogenic stroke and proven (asymptomatic) DVT (*N* = 5) the histological composition was still remarkable different from the composition of the DVT from our cohort (RBC 64% vs. 83%) again questioning whether DVT has any causality with PFO-associated stroke. The histology of DVT thrombi in our cohort also differed from that of thrombi defined as non-cardioembolic [7]. As described in the study by Hund et al., the non-cardioembolic group includes large artery atherosclerosis and other identified causes (e.g., carotid artery dissection). All with an arterial origin. Since DVT thrombi are formed in the venous vasculature and hence under lower flow, we anticipated that the histology of DVT thrombi would differ from that of non-cardioembolic clots.

The main limitation of our study is the small amount of thrombi available for analysis. This is largely due to the fact that we could only include patients who were identified with a clear PFO-associated stroke during initial admission, whereas most PFO-associated strokes are usually diagnosed later during outpatient clinic follow-up.

This is the first study to compare the composition of PFO-associated stroke thrombi with those derived from DVT and other cardioembolic causes of stroke. Further prospective studies into the histopathological aspects of thrombi and the cause of stroke are needed to optimize the personalized treatment strategies and to distinguish pathological PFOs from incidental PFOs.

## Conclusion

We observed that the composition of the three thrombi from patients with PFO-associated stroke differs from that of the four DVT thrombi in our cohort. Prospective studies are needed to determine whether thrombi in PFO-associated stroke share a similar pathophysiology with venous thrombi.

## Data Availability

The raw and anonymized data used in this study can be made available to other researchers on request. Written proposals can be addressed to the MR CLEAN Registry group.
